# Type I Interferon Signaling Is a Common Factor Driving Streptococcus pneumoniae and Influenza A Virus Shedding and Transmission

**DOI:** 10.1128/mBio.03589-20

**Published:** 2021-02-16

**Authors:** Tonia Zangari, Mila B. Ortigoza, Kristen L. Lokken-Toyli, Jeffrey N. Weiser

**Affiliations:** a Department of Microbiology, New York University Grossman School of Medicine, New York, New York, USA; b Department of Medicine, Division of Infectious Diseases, New York University Grossman School of Medicine, New York, New York, USA; Mississippi State University

**Keywords:** influenza virus, interferon, pneumococcus, transmission

## Abstract

The dynamics underlying respiratory contagion (the transmission of infectious agents from the airways) are poorly understood. We investigated host factors involved in the transmission of the leading respiratory pathogen Streptococcus pneumoniae. Using an infant mouse model, we examined whether S. pneumoniae triggers inflammatory pathways shared by influenza A virus (IAV) to promote nasal secretions and shedding from the upper respiratory tract to facilitate transit to new hosts. Here, we show that amplification of the type I interferon (IFN-I) response is a critical host factor in this process, as shedding and transmission by both IAV and S. pneumoniae were decreased in pups lacking the common IFN-I receptor (*Ifnar1*^−/−^ mice). Additionally, providing exogenous recombinant IFN-I to S. pneumoniae-infected pups was sufficient to increase bacterial shedding. The expression of IFN-stimulated genes (ISGs) was upregulated in S. pneumoniae-infected wild-type (WT) but not *Ifnar1*^−/−^ mice, including genes involved in mucin type O-glycan biosynthesis; this correlated with an increase in secretions in S. pneumoniae- and IAV-infected WT compared to *Ifnar1*^−/−^ pups. S. pneumoniae stimulation of ISGs was largely dependent on its pore-forming toxin, pneumolysin, and coinfection with IAV and S. pneumoniae resulted in synergistic increases in ISG expression. We conclude that the induction of IFN-I signaling appears to be a common factor driving viral and bacterial respiratory contagion.

## INTRODUCTION

Host-to-host transmission is a key step in the life cycle of infectious agents. Transmission involves exit from the colonized host (shedding), acquisition by a susceptible host, and establishment of colonization by the organism in the new host. Successful pathogens employ strategies such as inducing inflammation to promote their shedding via host processes. For highly contagious common respiratory tract infections, such as influenza, the common cold, or pertussis, disease is characterized by increased secretions that could promote both aerosol and contact routes of transmission. We have developed an infant mouse model to study shedding and transmission of viral and bacterial pathogens from the upper respiratory tract (URT) (1, 2), specifically the common URT pathogens Streptococcus pneumoniae and influenza A virus (IAV). While pneumococcal disease and colonization have been extensively studied in animal models, little is known about transmission of this common pathogen. Our studies on pneumococcal transmission have shown that a critical factor is the number of pneumococci shed from the nasopharynx of the infected host: high levels of shedding are correlated with higher intralitter transmission rates (1, 3), and induction of inflammation correlates with increased shedding (1, 4, 5). Expression of the S. pneumoniae toxin pneumolysin (Ply) has been shown to induce URT inflammation (6), leading to increased shedding and transmission (4). Similarly, levels of viral shedding, rather than titers in the URT, correlate with IAV transmission dynamics (2). S. pneumoniae colonization and disease are increased by recent or concurrent URT viral infection, especially with IAV (7, 8), and we have previously shown that coinfection with IAV promotes pneumococcal shedding in infant mice (1).

In the current study, we sought to better understand how URT colonization by S. pneumoniae promotes transmission. We hypothesized that S. pneumoniae usurps some of the same pathways as IAV to drive shedding via increased URT inflammation and secretions, thereby facilitating transmission to new hosts. We focused on IFN-I, as previous work in our laboratory has shown that, in an adult mouse model of S. pneumoniae colonization, pneumococcal infection alone was associated with an increase in expression of the IFN-I genes *Ifna* and *Ifnb* (9). A protective role for IFN-I against invasive pneumococcal disease has also been shown (10). Additionally, induction of IFN-I signaling was dependent on pore formation by Ply, which allows pneumococcal products to access cytosolic-sensing pathways and trigger proinflammatory responses (11, 12).

Here, we describe the stimulation of IFN-I signaling by S. pneumoniae as a critical host factor in our transmission model. We show that shedding and transmission of both IAV and S. pneumoniae are decreased in infant mice lacking the IFN-I receptor. IFN-stimulated genes (including genes involved in mucin biosynthesis) and mucus-containing secretions are upregulated in the URT mucosa of S. pneumoniae-infected mice. Our findings suggest a mechanism facilitating S. pneumoniae contagion that is shared by viral infection.

## RESULTS

### Type I interferon signaling is necessary for high-level pneumococcal and influenza shedding.

**FIG 1:**
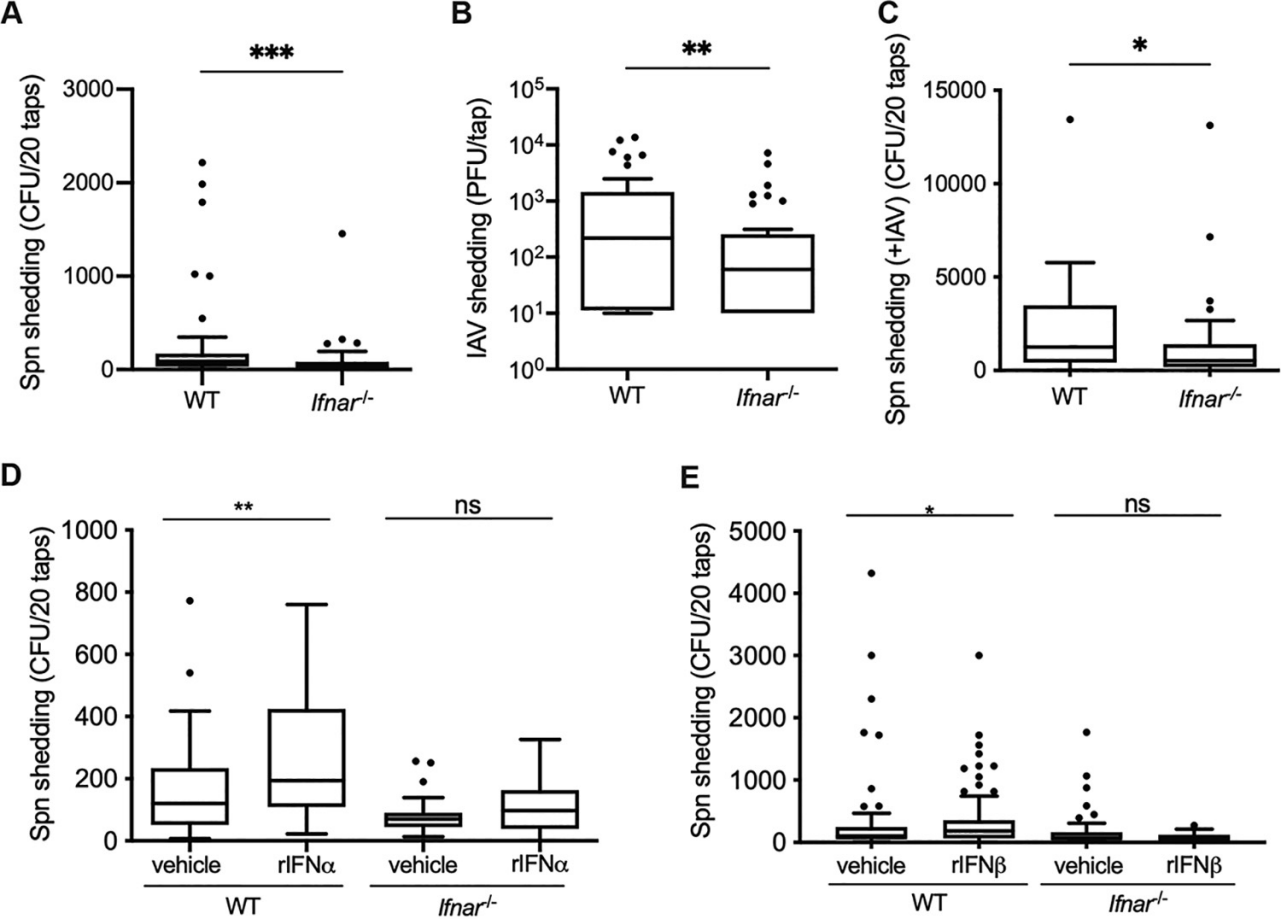
Type I interferon is necessary for and promotes high-level shedding of S. pneumoniae and IAV. WT and *Ifnar1*^−/−^ pups were infected i.n. with 10^3^ CFU S. pneumoniae (Spn), 250 PFU IAV-x31, or S. pneumoniae plus IAVx31. (A and B) *Ifnar1*^−/−^ pups shed significantly fewer bacteria than WT pups (A), and *Ifnar1*^−/−^ pups shed significantly less IAV than WT pups (B). (C) WT and *Ifnar1*^−/−^ pups first received IAV and then S. pneumoniae. *Ifnar1*^−/−^ pups shed significantly fewer bacteria than WT pups. (D and E) Exogenous recombinant IFN-α2 or IFN-β increase pneumococcal shedding from WT but not *Ifnar1*^−/−^ mice. WT and *Ifnar1*^−/−^ pups were infected i.n. with 10^3^ CFU S. pneumoniae and daily received either 1,000 IU of recombinant mouse IFN-α2 or 1,000 to 5,000 IU of recombinant mouse IFN-β or vehicle control (0.1% BSA-PBS) by i.n. instillation. Treatment of WT pups with rIFN-α2 (D) or rIFN-β (E) was sufficient to increase pneumococcal shedding over the baseline. Shedding data are shown as a Tukey box-and-whisker plot, with outliers shown as symbols. Each symbol represents the value from an individual pup on a single day. *n* ≥ 8 pups/group. ns, not significant; *, *P* < 0.05; **, *P* < 0.01; ***, *P* < 0.001 (Mann-Whitney test).

We utilized an infant mouse model (1, 3) to assess the effect of IFN signaling on colonization and shedding of S. pneumoniae. Wild-type (WT) and type I interferon receptor-deficient (*Ifnar1^−/−^*) pups were intranasally (i.n.) infected on day four of life with S. pneumoniae T4, and pneumococcal shedding from the nares of pups was enumerated on days 1 to 5 postinfection (p.i.), which we have determined to be the period of peak shedding (3). Colonization was measured on day 5 p.i.; upper respiratory tract (URT) samples were collected by lavage and plated to determine colonization density. Despite similar colonization levels (see Fig. S1A in the supplemental material), *Ifnar1*^−/−^ pups showed significantly lower S. pneumoniae T4 shedding (median, 30 CFU/mouse) than WT pups (median, 87.5 CFU/mouse; Fig. 1A), indicating a role for IFN-I signaling in S. pneumoniae shedding. As the association between influenza A virus (IAV) infection and stimulation of IFN-I signaling has long been established (reviewed in reference 13), we next assessed the role of IFN-I signaling in IAV (strain x31) shedding. WT and *Ifnar1^−/−^* pups were infected i.n. with IAV on days 4 to 7 of life, and shed virus was collected on days 1 to 4 p.i. The URT was lavaged on day 4 p.i., and samples were used to quantify virus via plaque assay. We found that IFN-I signaling is required for high-level IAV shedding (*Ifnar1*^−/−^ median, 60 PFU/mouse; WT median, 220 PFU/mouse; Fig. 1B), yet viral titers in the URT of infant mice were not different between the two mouse strains (Fig. S1B). Epidemiological studies have demonstrated that IAV infection increases transmission of S. pneumoniae (14, 15) (reviewed in reference 16), and we previously demonstrated that coinfection with IAV significantly increases S. pneumoniae shedding in our infant mouse model. To test whether loss of IFN-l signaling also impacts S. pneumoniae shedding during IAV coinfection, we i.n. infected WT and *Ifnar1*^−/−^ pups first with IAV and then with S. pneumoniae T4. Similar to what was observed during S. pneumoniae T4 single infection, coinfected *Ifnar1*^−/−^ pups (median, 526 CFU/mouse; Fig. 1C) shed significantly fewer S. pneumoniae T4 organisms than coinfected WT pups (median, 1,260 CFU/mouse; Fig. 1C) despite comparable S. pneumoniae T4 colonization levels in both mouse strains (Fig. S1C). Taken together, the shedding data indicate that IFN-I signaling promotes both bacterial and viral nasal shedding in the infant mouse model.

10.1128/mBio.03589-20.1 FIG S1 S. pneumoniae colonization and IAV titer do not differ in WT and *Ifnar1*^−/−^ pups. Pups were infected i.n. with either 10^3^ CFU S. pneumoniae or 250 PFU IAV-x31. (A) S. pneumoniae URT colonization was not different between WT and *Ifnar1*^−/−^ pups. (B) There was no difference in viral titer in the URT of WT and *Ifnar1*^−/−^ pups. (C) WT and *Ifnar1*^−/−^ pups first received IAV and then S. pneumoniae; bacterial colonization was not different between pups. (D and E) WT and *Ifnar1*^−/−^ pups were infected i.n. with 10^3^ CFU S. pneumoniae and daily received 1,000 IU of recombinant mouse IFN-α2 or 1,000 to 5,000 IU of recombinant mouse IFN-β or vehicle control (0.1% BSA-PBS) by i.n. instillation. Treatment of WT pups with rIFN-α2 (D) or rIFN-β (E) did not affect bacterial colonization. Colonization and titer data are for individual pups, with the line indicating the geometric mean. Each symbol represents the value from an individual pup on a single day. *n* ≥ 8 pups/group. ns, not significant (Mann-Whitney test). The dotted line shows the limit of detection. Download FIG S1, PDF file, 0.2 MB. Copyright © 2021 Zangari et al. 2021 Zangari et al. https://creativecommons.org/licenses/by/4.0/ This content is distributed under the terms of the Creative Commons Attribution 4.0 International license.

### Exogenous rIFN-α and rIFN-**β** increase pneumococcal shedding.

To further examine the effect of IFN-I on S. pneumoniae shedding, we i.n. administered recombinant mouse IFN-α2 or IFN-β daily to pups infected on day four of life with S. pneumoniae T4 and assessed pneumococcal shedding on days 1 to 5 p.i.; colonization was measured on day 5 p.i. In WT pups, treatment with recombinant IFN-α2 (rIFN-α2) (Fig. 1D) or rIFN-β (Fig. 1E) did not affect S. pneumoniae colonization (Fig. S1D and E) but resulted in increased S. pneumoniae T4 shedding compared to mice that received the vehicle control (Fig. 1D and E). This increase in pneumococcal shedding did not occur in *Ifnar1*^−/−^ mice, indicating a requirement for IFN-I signaling (Fig. 1D and E). Taken together, the data shown in Fig. 1 indicate that IFN-I signaling is necessary and sufficient to achieve high levels of pneumococcal shedding.

### S. pneumoniae is shed and stimulates IFN-I at 21 days p.i.

**FIG 2:**
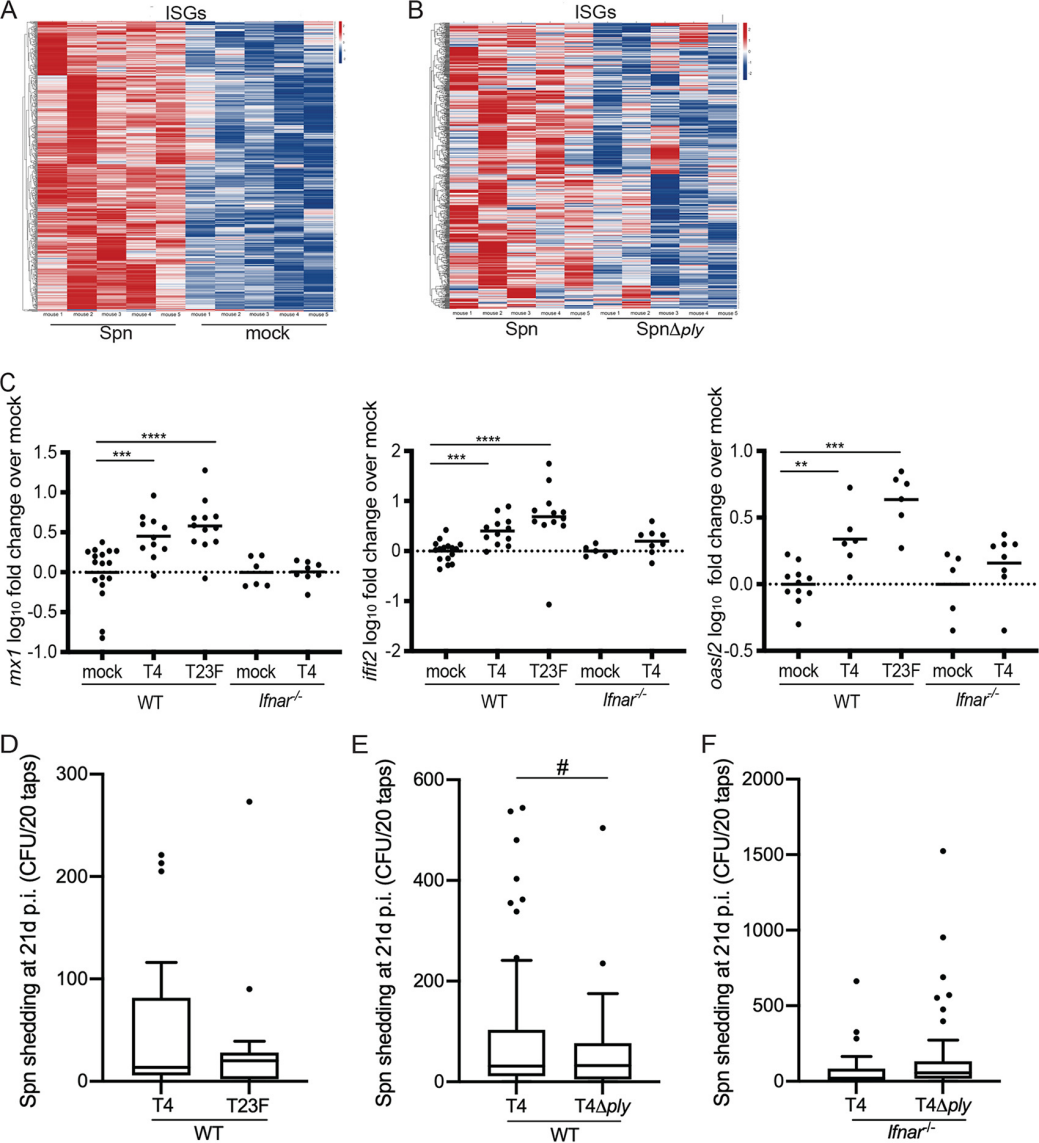
Pneumococci are shed and stimulate IFN-I at 21 days p.i. in a pneumolysin-dependent manner. (A and B) WT pups were infected i.n. with 10^3^ CFU S. pneumoniae; mock-treated mice received PBS. RNA was isolated from URT lavages on day 21 p.i. and analyzed by RNA-seq. Shown is a heat map of 543 genes identified as the interferome, comparing S. pneumoniae to mock infection (A) or S. pneumoniae to the S. pneumoniae *ply* mutant strain (B), demonstrating relative gene expression as log_2_ fold change, with increased expression in red and decreased expression in blue. *n* = 5 mice per group. (C) RNA was isolated from URT lavages on day 21 p.i. and analyzed by qRT-PCR. At 21 days p.i., S. pneumoniae-infected mice showed significantly increased expression of the ISGs *Ifit2*, *Mx1*, and *Oasl2* over mock-infected mice. This increased expression was seen only in WT and not *Ifnar1*^−/−^ mice. (D to F) WT pups were infected i.n. with 10^3^ CFU S. pneumoniae T4 or T23F (D), or WT and *Ifnar1*^−/−^ pups were infected i.n. with 10^3^ CFU S. pneumoniae T4 or an isogenic T4 *ply* mutant (E and F). (D) T4 and T23F are shed 21 days p.i., which correlates with gene expression data. (E) At 21 days p.i., there are more high-shedding events (>200) in S. pneumoniae-infected mice than mice infected with the isogenic S. pneumoniae *ply* mutant. (F) At 21 days p.i., there is no difference in the number of high-shedding events between S. pneumoniae and S. pneumoniae *ply* mutant strains in *Ifnar1*^−/−^ mice. Gene expression data are log_10_ transformed; each symbol represents the value from an individual pup, and the line represents the mean. Comparisons (Mann-Whitney test) are to mock-infected mice of the respective genotypes. Shedding data are shown as Tukey box-and-whisker plots, with outliers shown as symbols. Each symbol represents the value from an individual pup on a single day. *n* ≥ 8 pups/group. ns, not significant; **, *P* < 0.01; ***, *P* < 0.001; ****, *P* < 0.0001 (Mann-Whitney test); #, *P* = 0.03 (one-tailed Fisher’s exact test).

In contrast to viral infection, the effects of IFN-I and downstream stimulation of ISGs are not well documented for bacterial infection. We previously described an RNA-sequencing (RNA-Seq) screen that analyzed URT lavages collected from mice colonized with S. pneumoniae T23F for 21 days (pups were infected on day four of life [17]). Further analysis of T23F-infected mice compared to mock-infected mice at day 21 p.i. show increased expression of interferon-stimulated genes (ISGs) in the infected mice (Fig. 2A). Of the >900 genes with increased expression over mock infection at this time point (Table S1), 543 genes were annotated as IFN-I ISGs (Table S2), whereas only 128 of the 538 downregulated genes were considered ISGs (18). Closer analysis of *Ifit2*, *Mx1*, and *Oasl2*, three well-established ISGs, by quantitative reverse transcription-PCR (qRT-PCR) confirmed the increased expression in S. pneumoniae T4- and T23F-infected WT mice over mock-infected controls (Fig. 2C). In contrast, expression of *Ifit2*, *Mx1*, and *Oasl2* remained unchanged in S. pneumoniae-infected *Ifnar1*^−/−^ mice compared to controls (Fig. 2C). Considering the RNA-seq screen was performed using URT samples acquired during late S. pneumoniae infection (i.e., 21 days p.i.), we wanted to ensure that pneumococcal shedding continues at 21 days p.i. WT mice were infected as pups with S. pneumoniae; shedding was assessed to day 21 p.i., and colonization was determined following URT lavage on day 21 p.i. WT mice continued to shed S. pneumoniae to day 21 p.i. (Fig. 2D) and remained densely colonized with both T4 and T23F S. pneumoniae serotypes (Fig. S2A). Together, these results suggest that during late time points, S. pneumoniae colonization promotes IFN-l signaling and plays a role in enhancing pneumococcal shedding. However, it remains unclear how S. pneumoniae colonization drives IFN-l signaling and subsequent shedding.

10.1128/mBio.03589-20.2 FIG S2 S. pneumoniae T4 and T23F colonization at 21 days p.i. does not differ in WT and *Ifnar1*^−/−^ mice. (A to C) WT pups were infected i.n. with 10^3^ CFU S. pneumoniae T4 or T23F (A), or WT and *Ifnar1*^−/−^ pups were infected i.n. with 10^3^ CFU S. pneumoniae T4 or an isogenic T4 *ply* mutant; colonization was assessed 21 days p.i. (B and C). (A) The mice are still highly colonized with S. pneumoniae of both T4 and T23F serotypes. (B) Colonization of T4 and the isogenic T4 *ply* mutant strain is not different. (C) At 21 days p.i., there is no difference in the colonization between T4 and T4 *ply* mutant strains in *Ifnar1*^−/−^ mice. Colonization data are for individual pups, with the line indicating the geometric mean. Each symbol represents the value from an individual pup on a single day. *n* ≥ 8 pups/group. ns, not significant by Mann-Whitney test. Download FIG S2, PDF file, 0.2 MB. Copyright © 2021 Zangari et al. 2021 Zangari et al. https://creativecommons.org/licenses/by/4.0/ This content is distributed under the terms of the Creative Commons Attribution 4.0 International license.

10.1128/mBio.03589-20.5 TABLE S1 Genes upregulated in S. pneumoniae-infected mice over PBS mice at 21 days p.i. Mice received S. pneumoniae T23F or PBS, i.n., on day 4 of life; on day 21 p.i., the mice were euthanized and RNA was collected by URT lavage. Download Table S1, XLSX file, 0.1 MB. Copyright © 2021 Zangari et al. 2021 Zangari et al. https://creativecommons.org/licenses/by/4.0/ This content is distributed under the terms of the Creative Commons Attribution 4.0 International license.

10.1128/mBio.03589-20.6 TABLE S2 Interferome analysis of genes upregulated in S. pneumoniae-infected mice at 21 days p.i. The 915 genes identified in the RNA-seq were determined to be ISGs (*n* = 543) by use of the Interferome database, v2.01, at http://www.interferome.org/interferome/home.jspx (18). Download Table S2, XLSX file, 0.03 MB. Copyright © 2021 Zangari et al. 2021 Zangari et al. https://creativecommons.org/licenses/by/4.0/ This content is distributed under the terms of the Creative Commons Attribution 4.0 International license.

Previous work from our laboratory has shown that the presence of the S. pneumoniae toxin pneumolysin gene (*ply*) stimulated IFN-I in adult mice infected with T23F (9) and that *ply* correlated with higher T4 shedding in infant mice (4). Here, we extend those observations: further analysis from the 21-day time point of the RNA-seq screen revealed an overall reduction in the expression of genes involved in IFN-l signaling from mice infected with a *ply*-deficient S. pneumoniae strain compared to wild-type S. pneumoniae infection (Fig. 2B). To test whether the presence of pneumolysin enhances shedding through induction of IFN-l signaling during late infection, we infected WT and *Ifnar1*^−/−^ pups with either a *ply*-deficient S. pneumoniae T4 (T4Δ*ply*) or wild-type T4 strain and assessed shedding to day 21 p.i. We found that the impact of Ply on shedding is *Ifnar1* dependent: the contribution of *ply* to high S. pneumoniae shedding events (those of >200 CFU [4]) extends to day 21 p.i. (Fig. 2E), although mice infected with either S. pneumoniae T4 strain are colonized to the same level at 21 days p.i. WT mice shed T4 at higher levels than the T4Δ*ply* strain, a difference that was not seen in *Ifnar1*^−/−^ mice (Fig. 2F). Further, colonization levels of the wild-type S. pneumoniae T4 and T4Δ*ply* strains remained similar at day 21 p.i. in both WT and *Ifnar1*^−/−^ pups (Fig. S2B and C). Although the stimulation of ISGs was more robust with T23F than T4, this did not correlate with differences in Ply levels (Fig. S3). Together, these results suggest that pneumolysin drives pneumococcal shedding through stimulation of IFN-l signaling.

10.1128/mBio.03589-20.3 FIG S3 Immunoblot of S. pneumoniae strains for pneumolysin. S. pneumoniae serotypes T4 and T23F used in this study do not produce different levels of pneumolysin. Download FIG S3, PDF file, 0.4 MB. Copyright © 2021 Zangari et al. 2021 Zangari et al. https://creativecommons.org/licenses/by/4.0/ This content is distributed under the terms of the Creative Commons Attribution 4.0 International license.

### S. pneumoniae stimulates IFN-I early in infection.

**FIG 3:**
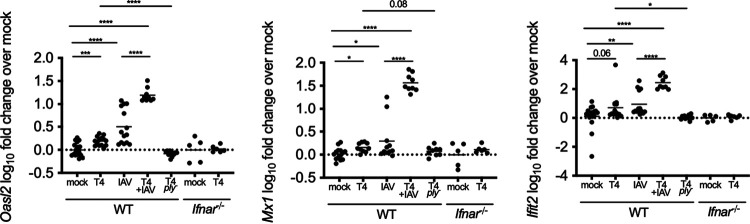
S. pneumoniae and IAV stimulate IFN-I signaling at 48 h p.i. WT and *Ifnar1*^−/−^ pups were infected i.n. with 10^3^ CFU the S. pneumoniae T4 or T4Δ*ply* mutant, 250 PFU IAV, or T4 plus IAV; mock-infected mice received PBS. RNA was isolated from URT lavages 48 h p.i. and analyzed by qRT-PCR. S. pneumoniae-infected mice showed significantly increased expression of the ISGs *Ifit2*, *Mx1*, and *Oasl2* compared to that of mock-infected mice. This increased expression was seen only in WT and not *Ifnar1*^−/−^ mice. The stimulation of ISGs by S. pneumoniae was *ply* dependent. Gene expression data are log_10_ transformed; each symbol represents the value from an individual pup. Comparisons (Mann-Whitney test) are to mock-infected mice of the respective genotype. *n* ≥ 5 pups/group. ns, not significant; *, *P* < 0.05; **, *P* < 0.01; ***, *P* < 0.001; ****, *P* < 0.0001.

Figures 1 and 2 show that shedding of S. pneumoniae is dependent on IFN-l signaling. However, it remained unclear if the increase in expression of ISGs identified from late infection was also induced during early infection. Expression of *Ifit2*, *Mx1*, and *Oasl2* was assessed at an early time point (48 h p.i.) in WT and *Ifnar1*^−/−^ mice (Fig. 3A). Four-day-old pups were infected i.n. with the S. pneumoniae T4 or T4Δ*ply* strain or received phosphate-buffered saline (PBS). In WT but not *Ifnar1*^−/−^ mice, S. pneumoniae colonization induced increased *Ifit2*, *Mx1*, and *Oasl2* expression over uninfected control mice, and this was *ply* dependent (Fig. 3A). In contrast to previous studies in adult mice (9), we were unable to detect increased expression of *Ifna* or *Ifnb* in S. pneumoniae-infected mice at this time point (Fig. S4A and B). To assess if S. pneumoniae and IAV stimulate similar ISGs through the IFN-I pathway, we infected pups with IAV-x31 or T4 plus IAV (coinfected pups received IAV the day prior to S. pneumoniae infection, so the time point remains consistent as 48 h after S. pneumoniae infection). As expected, IAV infection also resulted in increased expression of *Ifit2*, *Mx1*, and *Oasl2* over mock infection (Fig. 3). Of note, *Ifit2*, *Mx1*, and *Oasl2* expression is increased in T4 plus IAV-coinfected mice over that in mice infected with only IAV or S. pneumoniae, indicating a synergistic role of S. pneumoniae in inducing IFN-I in mice that are infected with IAV. Together, these data indicate that both bacterial and viral URT infections stimulate ISGs in the infant mouse model.

10.1128/mBio.03589-20.4 FIG S4 Increased expression of *Ifna* and *Ifnb* was not detected at 48 h p.i. in mice that had received S. pneumoniae. WT pups were infected i.n. with 10^3^ CFU S. pneumoniae T4 or 250 PFU IAV; mock-infected mice received PBS. RNA was isolated from URT lavages 48 h p.i. and analyzed by qRT-PCR. S. pneumoniae-infected mice showed no increased expression of *Ifna* or *Ifnb* over mock-infected mice. This increased expression was seen only in IAV-infected mice. Gene expression data are log_10_ transformed; each symbol represents the value from an individual pup. Comparisons (Mann-Whitney test) are to mock-infected mice. *n* ≥ 5 pups/group. ns, not significant; **, *P* < 0.01. Download FIG S4, PDF file, 0.2 MB. Copyright © 2021 Zangari et al. 2021 Zangari et al. https://creativecommons.org/licenses/by/4.0/ This content is distributed under the terms of the Creative Commons Attribution 4.0 International license.

### S. pneumoniae colonization increases nasal secretions of pups.

**FIG 4:**
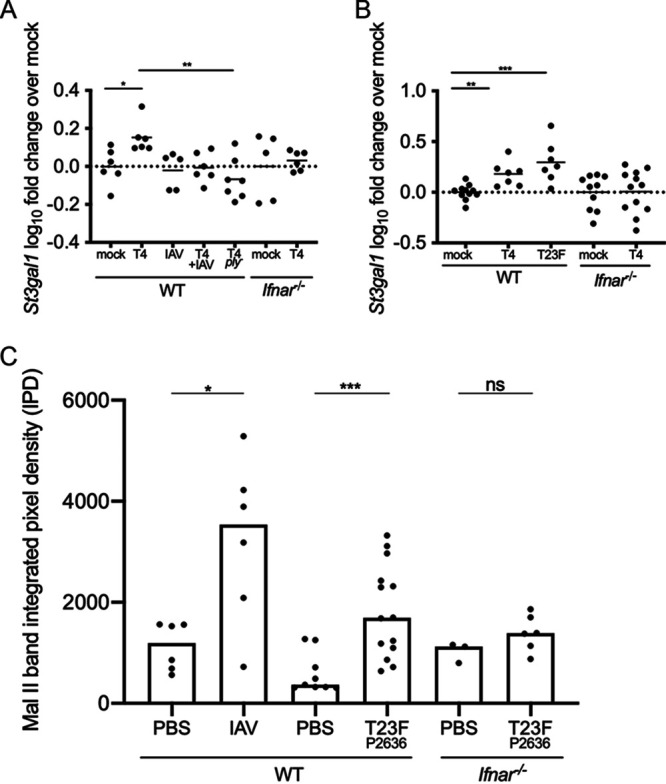
S. pneumoniae colonization increases nasal secretions of pups. (A and B) WT and *Ifnar1*^−/−^ pups were infected i.n. with 10^3^ CFU S. pneumoniae or 250 PFU IAV; mock-infected mice received PBS. RNA was isolated from URT lavages 48 h (A) or 21 days (B) p.i. and analyzed by qRT-PCR. S. pneumoniae-infected mice showed significantly increased expression of the ISG sialyltransferase *St3gal1* over mock-infected mice. This increased expression was seen only in WT and not *Ifnar1*^−/−^ mice. The stimulation of ISGs by S. pneumoniae was also *ply* dependent. Gene expression data are log_10_ transformed; each symbol represents the value from an individual pup, and the line represents the mean. Comparisons (Mann-Whitney test) are to mock-infected mice of the respective genotype. *n* ≥ 5 pups/group. ns, not significant; *, *P* < 0.05; **, *P* < 0.01; ***, *P* < 0.001. (C) Pups infected with IAV or S. pneumoniae secreted more sialic acid from their nares than pups that received PBS. IAV comparisons are days 14 to 18 p.i. to matched PBS controls; S. pneumoniae comparisons are of days 9 to 12 p.i. The increase in nasal secretions from S. pneumoniae-infected pups is IFN-I dependent, as there was no difference detected in sialic acid shed from infected *Ifnar1*^−/−^ pups compared to PBS controls (days 9 to 12 p.i.). WT and *Ifnar1*^−/−^ pups were infected i.n. with either 10^3^ CFU S. pneumoniae T23F P2636 (neuraminidase mutant) or 250 PFU IAV-x31 or received PBS. Band integrated pixel density data are for individual pups, with the bar indicating the median. Each symbol represents the value from an individual pup on a single day. *n* ≥ 3 pups/group. ns, not significant; *, *P* < 0.05; **, *P* < 0.01; ***, *P* < 0.001 (Mann-Whitney test).

Rhinorrhea is associated with colonization and transmission of S. pneumoniae in children (14, 19), and we have hypothesized that S. pneumoniae colonization results in increased secretions in our infant mouse model (1, 3, 4). To this end, we analyzed the RNA-seq screen and noted increased expression of multiple genes involved in mucin type-O glycan biosynthesis during S. pneumoniae infection at day 21 p.i. (Table S1). Mucins are composed of threadlike core proteins that contain O-linked oligosaccharide chains that extend outward and are the main glycans of mucus polymers (20–23). Specifically, *St3gal1* (ST3 beta-galactosidase alpha-2,3-sialyltransferase 1, which adds a sialic acid in an α2,3 linkage to Galβ1,3 GalNAc) was identified as part of the interferome of genes upregulated in S. pneumoniae-infected mice (Table S2). We confirmed the increased expression of *St3gal1* in S. pneumoniae-infected WT mice at 48 h p.i. (Fig. 4A) and on day 21 p.i. (Fig. 4B) by qRT-PCR. Interestingly, at 48 h p.i., only pneumococcal colonization results in increased *St3gal1* expression, indicating that it is an ISG stimulated by bacterial but not viral infection. The induction of *St3gal1* was confirmed to be dependent on IFN-l signaling, as expression of *St3gal1* from S. pneumoniae-infected *Ifnar1*^−/−^ mice remained unchanged compared to that of mock mice at 48 h p.i. (Fig. 4A) and 21 days p.i. (Fig. 4B). Similar to the other ISGs analyzed, the increased expression of *St3gal1* in T4-infected mice was dependent on the presence of *ply* (Fig. 4A).

To determine if the upregulation of mucin biosynthesis genes observed in mice infected with S. pneumoniae correlated with increased URT secretions, we collected nasal taps from pups infected with S. pneumoniae or IAV and analyzed the samples by immunoblotting. The nares of pups were tapped into Tris-buffered saline (TBS), which was applied to nitrocellulose by vacuum in a slot blot minifold. The blot was incubated with Maackia Amurensis lectin II (Mal II), which recognizes α-2,3-linked sialic acids. IAV infection resulted in more nasal secretions, as assessed by the amount of sialic acid, than PBS treatment (Fig. 4C), and, similarly, comparisons of the pixel density of bands from S. pneumoniae-infected mice to PBS-treated mice showed increased sialic acid secretions from S. pneumoniae mice (Fig. 4C). Further, this effect was IFN-I dependent, as there was no difference in sialic acid secretions from *Ifnar1*^−/−^ pups infected with S. pneumoniae compared to PBS treatment (Fig. 4C).

### Absence of IFN-I signaling decreases transmission of S. pneumoniae and IAV-x31.

**FIG 5:**
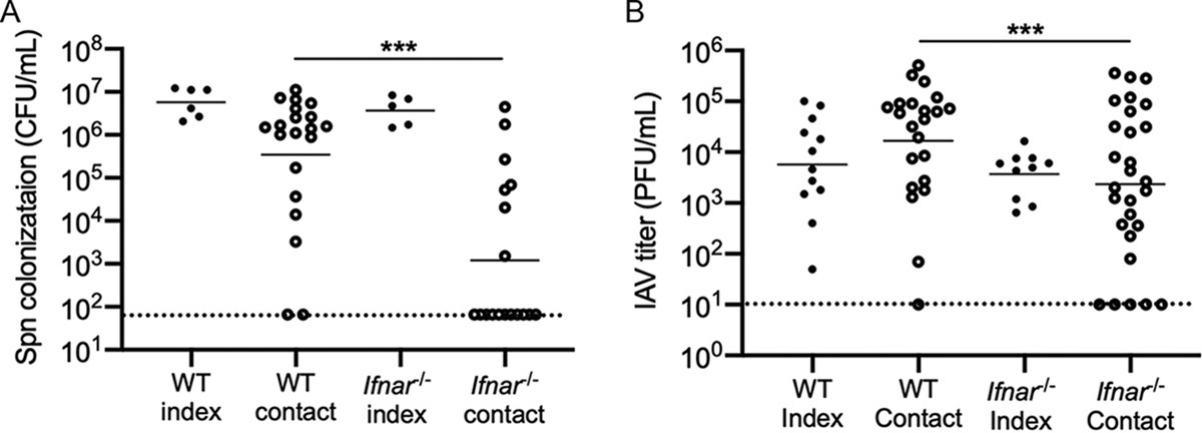
Lack of IFN-I signaling reduces transmission of IAV and S. pneumoniae. (A) WT and *Ifnar1*^−/−^ index pups were infected i.n. with 10^3^ CFU S. pneumoniae at a ratio of 6 index to 20 contact in WT litters or 5 index to 18 contact in *Ifnar1*^−/−^ mice in three separate litters. The S. pneumoniae transmission rate and bacterial burden were higher in WT than *Ifnar1*^−/−^ contact mice. (B) WT and *Ifnar1*^−/−^ index pups were infected i.n. with 250 PFU IAV at a ratio of 12 index to 22 contact in WT litters or 10 index to 28 contact in *Ifnar1*^−/−^ mice in five separate litters. The IAV titer was higher in WT than *Ifnar1*^−/−^ contact mice. Colonization and titer data are for individual pups, with the line indicating the geometric mean. Each symbol represents the value from an individual pup on a single day. *n* ≥ 5 pups/group. *, *P* < 0.05; ***, *P* < 0.001 (Mann-Whitney test). The dotted line shows the limit of detection.

Finally, we addressed the role of IFN-I signaling in close-contact transmission of S. pneumoniae from one infected pup to a susceptible littermate. We utilized a previously described model of S. pneumoniae transmission (1) in which we examined pneumococcal transmission in the setting of IAV-x31 coinfection, as S. pneumoniae transmission is amplified by IAV coinfection (whereas the low rate of S. pneumoniae transmission in a monoinfection model is insensitive for the detection of factors that partially reduce transmission). “Index” mice were infected with T4, and the remaining pups in the litter were not infected (“contact” mice). Four days later, all pups received IAV-x31. After a further 5-day exposure period (9 days after S. pneumoniae infection), all pups were euthanized and assessed for pneumococcal colonization. Each colonized contact pup was considered a transmission event. Transmission of S. pneumoniae T4 in WT litters was compared to transmission within *Ifnar*^−/−^ litters. The loss of IFN-I signaling decreased S. pneumoniae transmission from 92.9% to 38.9% (Fig. 5A). Using a model of IAV transmission (2), we next assessed the impact of IFN-I on IAV-x31 transmission. Approximately one-third of a litter was infected with IAV on day 4 of life, and on day 4 p.i. all pups were euthanized and assessed for URT viral titer. In *Ifnar1*^−/−^ contact pups, the IAV titer was significantly lower than that in WT contact pups (Fig. 5B). The lower titer in *Ifnar^−/−^* contact pups indicated delayed transmission kinetics. These data indicate that IFN-I signaling promotes transmission of both S. pneumoniae and IAV, likely by promoting exit (shedding) of the pathogens from the infected host.

## DISCUSSION

Host factors contributing to respiratory contagion (the transmission of infectious agents from the airways) are poorly understood (24). Our tractable infant mouse models of respiratory transmission of S. pneumoniae and influenza A virus have allowed for the identification of common pathways facilitating their transit from host to host (2, 3). A caveat of our study is that, in our model, we are unable to distinguish contact versus aerosol routes of transmission within litters. URT infection of infant mice by both pathogens was shown to induce IFN-I responses, and, in the absence of IFN-I signaling (*Ifnar1*^−/−^ mice lacking the common IFN-α/β receptor), transmission of both S. pneumoniae and IAV was reduced. Prior studies demonstrate that numbers of bacteria or virions shed from the upper respiratory tract correlate with the rate of pup-to-pup transmission and that shedding levels correspond to the inflammatory response in the URT mucosa (1, 2, 4). Inflammation increases the production and flow of mucus secretions that drive shedding and contribute to transmission via contact or aerosol routes (which cannot be distinguished in this animal model). This study documents the contribution of IFN-I signaling to these events.

In contrast to the rhinorrhea that accompanies influenza during natural infection of humans, S. pneumoniae colonization is generally considered asymptomatic, although in young children there is a correlation between rhinitis and S. pneumoniae carriage (25). In adults, colonization is not associated with nasal symptoms, perhaps explaining why children are generally considered the main source of S. pneumoniae contagion (26). Additionally, young children are more commonly colonized and have an increased burden of organisms compared to adults. Further, high-density nasal carriage of pathogenic bacteria in childhood is transient and often associated with a URT viral infection (27). Similarly, in our infant mouse model, shedding dynamics correspond to the marked acute inflammatory response that typically characterizes IAV infection (1). In the current study, the IFN-I response to IAV was more robust than the response to S. pneumoniae, while the effects of coinfection with S. pneumoniae plus IAV on ISG expression were synergistic, potentially explaining the greatly increased S. pneumoniae shedding and transmission in the setting of IAV coinfection. These observations concur with those of an experimental human pneumococcal carriage study, in which S. pneumoniae colonization of adults that had been vaccinated with live attenuated influenza virus (LAIV) showed increased expression of ISGs in the URT epithelium compared to participants that were LAIV positive and S. pneumoniae carriage negative (28).

We show here that, in our mouse model, ISGs were expressed as early as 48 h after S. pneumoniae infection and were still expressed 21 days later, a finding that correlates with the continued shedding of the bacteria at this time point. The effect of S. pneumoniae colonization on the sustained expression of ISGs, annotated as the “interferome” (18), was extensive, encompassing approximately two-thirds of the upregulated genes at day 21 postinfection (see Table S2 in the supplemental material). In contrast to the stimulation of genes involved in IFN-I responses, 76 IFN-II ISGs (and 361 shared IFN-I/IFN-II ISGs) and zero IFN-III ISGs showed increased expression. It remains unclear how these responses and IFN-I signaling act to promote nasal secretions, although among the number of ISGs induced by S. pneumoniae colonization were those involved in the biosynthesis of O-linked glycans, which is a major component of mucin glycoproteins (23). Although it has been reported that there is increased expression of the major secretory mucins (e.g., *Muc5ac*) in the URT of adult mice coinfected with S. pneumoniae and IAV (8, 29), in the current study we were unable to document increased gene expression of the same respiratory mucins during S. pneumoniae colonization, perhaps because of the limited sensitivity of measuring transcriptional changes in the URT mucosa of pups as young as 6 days of age.

In this study, we found that in the absence of the IFN-I receptor, neither shedding nor transmission was completely eliminated, which suggests that other host pathways must also contribute to these processes. While *Ifnar1*^−/−^ mice lack the capacity for autocrine and paracrine amplification of signaling downstream of IFN-α and IFN-β due to the absence of the IFN-α/β receptor, intracellular triggering of IFN-I and ISGs remains intact. It is possible that the downstream effects of this signaling contribute to low-level shedding and transmission of S. pneumoniae and IAV. Of note, we did not detect increased expression of either *Ifna* or *Ifnb* in response to S. pneumoniae colonization of infant mice; possibly these responses are transient or early stimulation of IFN-I is relatively mild in pups (Fig. S3A and B). During experimental human pneumococcal colonization studies in healthy adults, carriage was associated with increased *IFNA* and *IFNB* gene expression in the respiratory epithelium (30).

The relationship between increased IFN-I expression, increased acute inflammation, and high levels of shedding are demonstrated by the altered responses when *Tlr2^−/−^* mice are infected with IAV and S. pneumoniae: coinfected *Tlr2*^−/−^ pups showed a significant reduction in *Ifna* transcript compared to WT pups 14 days p.i. (1, 31). In the case of S. pneumoniae monoinfection, *in vitro* and *in vivo* studies show that S. pneumoniae triggers IFN-I in a Ply-dependent manner (9, 32). Following uptake by professional phagocytes, this pore-forming toxin allows bacterial PAMPs to access the cytosol; cytosolic sensing and inflammatory responses lead to the production of IFN-I and stimulation of ISGs in a manner that may overlap cellular responses to viral infection (11, 12). Our observations here linking Ply and IFN-I signaling could account for the previously described reduction in high-density S. pneumoniae shedding during infection by *ply*-deficient mutant strains (4).

Further elucidation of how specific ISGs work together to modulate the volume and/or characteristics of respiratory secretions could provide a framework for defining the mechanisms leading to viral and bacterial shedding and transmission.

## MATERIALS AND METHODS

### Bacterial strains and culture.

Streptococcus pneumoniae strains were grown in tryptic soy (TS) broth (BD, Franklin Lakes, NJ) at 37°C without aeration to an optical density at 620 nm (OD_620_) of 1.0, or incubated on TS agar plates supplemented with 100 μl of catalase (30,000 U/ml; Worthington Biochemical) and streptomycin (Str; 200 μg/ml) or kanamycin (Kan; 125 μg/ml) at 37°C in 5% CO_2_ overnight. S. pneumoniae isolate P2406 is an Str^r^ derivative of TIGR4 (capsule type 4, T4 [3]). Strain P2499 is an Str-resistant derivative of P1121, a capsule type 23F (T23F) strain isolated from the nasopharynx in a human experimental-carriage study (33). Strain P2424, Str^r^, is a pneumolysin (*ply*)-deficient mutant of P2406 (4). Strain P2636, used in the immunoblot assay of nasal secretions, is serotype 23F in which *nanA* is deleted and *nanB* is disrupted by the Janus cassette; the strain is Str^s^, Kan^r^.

### IAV strains and culture.

A/X-31 (H3N2) virus (with HA/NA genes from A/Aichi/2/1968 and internal genes from A/Puerto Rico/8/1934) was a gift from Jan Erickson (University of Pennsylvania). Madin-Darby canine kidney (MDCK) cells were cultured in Dulbecco’s modified Eagle’s medium (high glucose, pyruvate; 11995065; Gibco) with 10% fetal bovine serum (PS-FB1; Peak Serum) and 1% penicillin-streptomycin (15070063; Gibco). Viral titers were determined by standard plaque assay in MDCK cells in the presence of TPCK (tosylsulfonyl phenylalanyl chloromethyl ketone)-treated trypsin (Thermo Scientific) (34). Virus was prepared as described previously (2). Briefly, IAV was propagated in 8- to 10-day-old embryonated chicken eggs (Charles River, CT) for 2 days at 37°C. Allantoic fluid from eggs containing virus was collected, followed by centrifugation (3,000 × *g*, 30 min, 4°C) to remove debris, aliquoted, and stored at −80°C.

### Mouse studies.

Wild-type C57BL/6J (strain 000664; Jackson Laboratories, Bar Harbor, ME) or *Ifnar1*^−/−^ mice lacking the type I interferon receptor (strain 028288; Jackson Laboratories) were used in all experiments. Mice were maintained and bred in a conventional animal facility. Pups were housed with a dam for the course of the experiments, until 3 to 4 weeks of age. Following infection, all mice appeared healthy, showed normal activity, and gained weight similarly to uninfected controls. These studies were conducted in accordance with the recommendations of the *Guide for the Care and Use of Laboratory Animals* (35). All mouse studies were approved by the Institutional Animal Care and Use Committee of the New York University Medical Center. All procedures were in compliance with the *Biosafety in Microbiological and Biomedical Laboratories* (https://www.cdc.gov/labs/pdf/CDC-BiosafetyMicrobiologicalBiomedicalLaboratories-2020-P.pdf).

### S. pneumoniae shedding and colonization.

Four-day-old pups were infected with 10^3^ CFU of S. pneumoniae in 3 μl of PBS by intranasal (i.n.) instillation with a blunt pipette tip, without anesthesia, and returned to their dam for the duration of the experiment. To assess daily nasal shedding of S. pneumoniae, the nose of the pup was gently tapped 20 times evenly across a TS-Str plate; the sample was spread with a sterile cotton swab and the colonies were enumerated after overnight incubation. Due to variation in day-to-day shedding, values collected daily over the entire infection period were combined for statistical analysis to allow for meaningful comparisons. At the end of the study, pups were euthanized by CO_2_ asphyxiation and cardiac puncture. Upper respiratory tract (URT) colonization of S. pneumoniae was determined as described previously (1). Briefly, the trachea was lavaged with 200 μl of sterile PBS, which was collected from the nose. Tenfold serial dilutions of the lavage sample were plated to TS-Str and enumerated after overnight incubation.

### Influenza A virus shedding and titers.

Pups (4 to 7 days of age) were infected by i.n. instillation with 250 PFU of IAV-x31 in 3 μl PBS, without anesthesia, and returned to the dam for the duration of the experiment. Shed virus was collected by dipping the nares of each mouse once into viral medium (PBS plus 0.3% bovine serum albumin [BSA]) daily, and samples were evaluated via plaque assay in MDCK cells. The pups were euthanized by CO_2_ asphyxiation followed by cardiac puncture. The URT was lavaged with 300 μl PBS collected through the nares, and samples were used to quantify virus via plaque assay.

### Intranasal treatment with recombinant IFN-I.

One day prior to S. pneumoniae infection and daily during the course of the experiment, litters were divided and half of the pups were treated i.n. with 1,000 IU of recombinant mouse IFN-α2 (CK83-10ug_NP; Novoprotein) or 1,000 to 5,000 IU of recombinant mouse IFN-β (8234-MB; R&D Systems) in 3 μl, and the other half of the litter received 3 μl of a vehicle control (0.1% BSA-PBS). Pneumococcal shedding was assessed 24 h after each IFN treatment for 5 days.

### S. pneumoniae transmission.

Pneumococcal transmission among pups was carried out as previously described (1). Four-day-old pups were infected with 10^3^ CFU of S. pneumoniae in 3 μl of PBS by i.n. instillation and returned to the litter (“index” pups). The ratio of index to uninfected “contact” pups was 1:3 to 1:4 in each litter. Four days later, all pups in the litter received IAV-x31 in 3 μl of PBS by i.n. instillation. Five days post-IAV (9 days after S. pneumoniae), the pups were euthanized and a URT lavage was done to quantify S. pneumoniae colonization of all pups.

### Influenza A virus transmission.

IAV transmission among pups was carried out as previously described (2). Four- to 7-day-old pups were infected by i.n. instillation with 250 PFU of IAV-x31 in 3 μl PBS and returned to the litter (index pups). The ratio of index to uninfected contact pups was 1:3. Intralitter transmission was determined at 4 days p.i.: pups were euthanized, and a URT lavage was done to quantify IAV titers of all pups.

### RNA-sequencing and analysis.

The RNA-sequencing experiment was previously described (17). Briefly, 4-day-old pups were intranasally infected, as described above, with S. pneumoniae T23F or its *ply* mutant or received PBS. Twenty-one days p.i., pups were euthanized by CO_2_ asphyxiation and cardiac puncture. URT colonization of S. pneumoniae was determined by lavage with 200 μl PBS, which was collected from the nose. Tenfold serial dilutions of the lavage sample were plated to TS-Str and enumerated after overnight incubation. Following the PBS lavage, a second URT lavage with 600 μl RLT lysis buffer was collected. RNA was extracted according to the manufacturer’s directions (RNeasy kit; Qiagen), and five replicates per group were subjected to RNA sequencing (RNA-seq) using Hi-seq. The raw fastq reads were aligned to the mm10 mouse reference genome using STAR aligner (36). The Illumina TruSeq stranded mRNA library prep kit (20020594; Illumina) was used to prepare libraries (input of 350 ng/sample with 11 cycles of final amplification). Fastq Screen was used to check for any contaminations in the samples, and Picard RNA-seqMetrics was used to obtain the metrics of all aligned RNA-seq reads. *featureCounts* (37) was used to quantify the gene expression levels. The raw gene count data were used for further differential expression analysis. To identify the differentially expressed genes (DEGs), the *DESeq2* R package (38) was used. The resulting genes with a false discovery rate (FDR) of <0.05 were considered significant. Heatmaps were generated using the *pheatmap* R package.

Genes identified in the RNA-seq were determined to be ISGs by use of the Interferome database, v2.01, at http://www.interferome.org/interferome/home.jspx (18).

### qRT-PCR.

The trachea of mice were first lavaged with 200 μl of sterile PBS, collected from the nose, to determine bacterial colonization. They were then lavaged with 600 μl of buffer RLT (79216; Qiagen) plus 1% β-mercaptoethanol (M3148; Sigma-Aldrich); samples were stored at −80°C until processing. RNA extraction (74106; RNeasy; Qiagen) and cDNA generation (4368814; high-capacity cDNA reverse transcriptase kit; Applied Biosystems, Thermo Fisher Scientific) were done per the manufacturers’ instructions. qRT-PCRs were run with Power Sybr green master mix (4368577; Applied Biosystems, Thermo Fisher Scientific), contained ∼10 ng cDNA and 0.5 μM primers (Table S3), and were run in a 384-well plate (Bio-Rad) using the CFX384 real-time system (Bio-Rad). Samples were run in duplicate, and all comparisons were run on the same plate. The expression of *gapdh* was an internal control, and fold change in gene expression was quantified according to the ΔΔ*C_T_* method (39).

10.1128/mBio.03589-20.7 TABLE S3 Primers (forward and reverse) used in RT-PCR analysis of the expression of interferon stimulated genes (plus *Gapdh* control) from the mouse upper respiratory tract. Download Table S3, DOCX file, 0.01 MB. Copyright © 2021 Zangari et al. 2021 Zangari et al. https://creativecommons.org/licenses/by/4.0/ This content is distributed under the terms of the Creative Commons Attribution 4.0 International license.

### Immunoblot of nasal secretions.

An assessment of α-2,3-linked sialic acid shed from the nares of pups was done by immunoblotting. Four-day-old pups were infected with S. pneumoniae as described above or received PBS, or 7- to 9-day-old pups received IAV as described above or received PBS (IAV pups were infected at an older age so they would survive the duration of the study). In this experiment, we utilized a T23F strain in which the neuraminidase genes *nanA* and *nanB* had been interrupted to avoid the confounding effects of these glycosidases on the assay. To assess daily nasal shedding, the nose of the pup was gently tapped 5 times into 350 μl Tris-buffered saline (TBS) in the bottom of a 6-well plate; the sample was collected and stored at −20°C. Slot blots were prepared on a 72-well Minifold II slot-blot apparatus (Schleicher & Schuell). Nasal shedding samples (300 μl) were applied to a 0.2-μm nitrocellulose membrane (0.2 μm; GE10600094; Amersham Protran) with vacuum. A sample collected on day 0 (before infection) was included on every membrane as a background control, and a 200-μl lavage sample was included on every membrane as a standard to allow for comparisons across blots. After sample application, the membrane was removed from the apparatus, air dried briefly, and incubated in 2% BSA-TBS at 4°C overnight. The membrane was incubated for 1 h at room temperature in 2 μg/ml Maackia Amurensis lectin II biotinylated (B-1265; Mal II; Vector Laboratories) diluted in TBS and was then washed 6 times (5 min each wash) with 0.1% Tween 20-TBS and incubated for 1 h at room temperature with streptavidin-horseradish peroxidase (HRP) conjugate (21130; Pierce) diluted 1:100,000 in TBS. The membrane was washed once for 1.5 h and then 5 times (15 min each wash) with 0.1% Tween 20–TBS. The membrane was developed with the SuperSignal West Femto substrate (34095; Thermo Scientific) according to the manufacturer's directions. Chemiluminescent signal was visualized, and the relative intensities of the bands on the blot were quantified by assessing integrated pixel density using the iBright CL1000 imaging system (ThermoFisher Scientific).

### Immunoblot for pneumolysin.

S. pneumoniae was grown to an OD_620_ of 0.5 in TS. Aliquots (1 ml) were spun down and resuspended in 83 μl of SDS loading buffer and were boiled for 10 min. Samples (20 μl, 1×, or 40 μl, 2×) were run on a 4-12% Bis-Tris gel (NW04122BOX; Invitrogen) in 1× morpholineethanesulfonic (MES) acid SDS running buffer (B0002; Invitrogen) at 150 V. Protein was transferred to a nitrocellulose membrane using the iBlot 2 dry blotting system (7-min transfer program P0; IB21001; Invitrogen). The membrane was then washed in Tris-saline blotting buffer (TSBB; 10 mM Tris-HCl [pH 8.0], 0.5 M NaCl, 0.5% Tween 20, and 0.02% sodium azide) for 2 min at room temperature and blocked in 1% BSA-PBS with shaking for 2 h at room temperature, after which anti-pneumolysin primary antibody (clone 1F11; HYB0410102; Invitrogen) was added (at a 1:1,000 dilution in 1% BSA-PBS) at 4°C overnight, with shaking. The membrane was washed in TSBB 3 times for 5 min each time at room temperature and incubated in goat anti-mouse IgG-AP (1:30,000 dilution in 1% BSA-PBS; A5153; Sigma) for 2 h at room temperature. The membrane was washed in TSBB 3 times for 5 min each time at room temperature before developing with the 5-bromo-4-chloro-3-indolylphosphate–nitroblue tetrazolium substrate (34042; Thermo Scientific) according to the manufacturer’s instructions.

### Statistical analysis.

Statistical analysis was done using GraphPad Prism 6.0 (GraphPad Software, San Diego, CA, USA). Unless indicated otherwise, differences between two groups were compared using Mann-Whitney test, where a *P* value of ≤0.05 was considered significant.

### Data availability.

RNA-seq data are available in the GEO repository under project accession number GSE116604.
