# Supplementary material for: Type I Interferon Signaling Is a Common Factor Driving Streptococcus pneumoniae and Influenza A Virus Shedding and Transmission
Source: mBio. 2021 Feb 16;12(1):e03589-20. doi: 10.1128/mBio.03589-20 (PMC8545127; doi:10.1128/mBio.03589-20)
Supplement: FIG S1 [file mbio.03589-20-sf001.pdf]

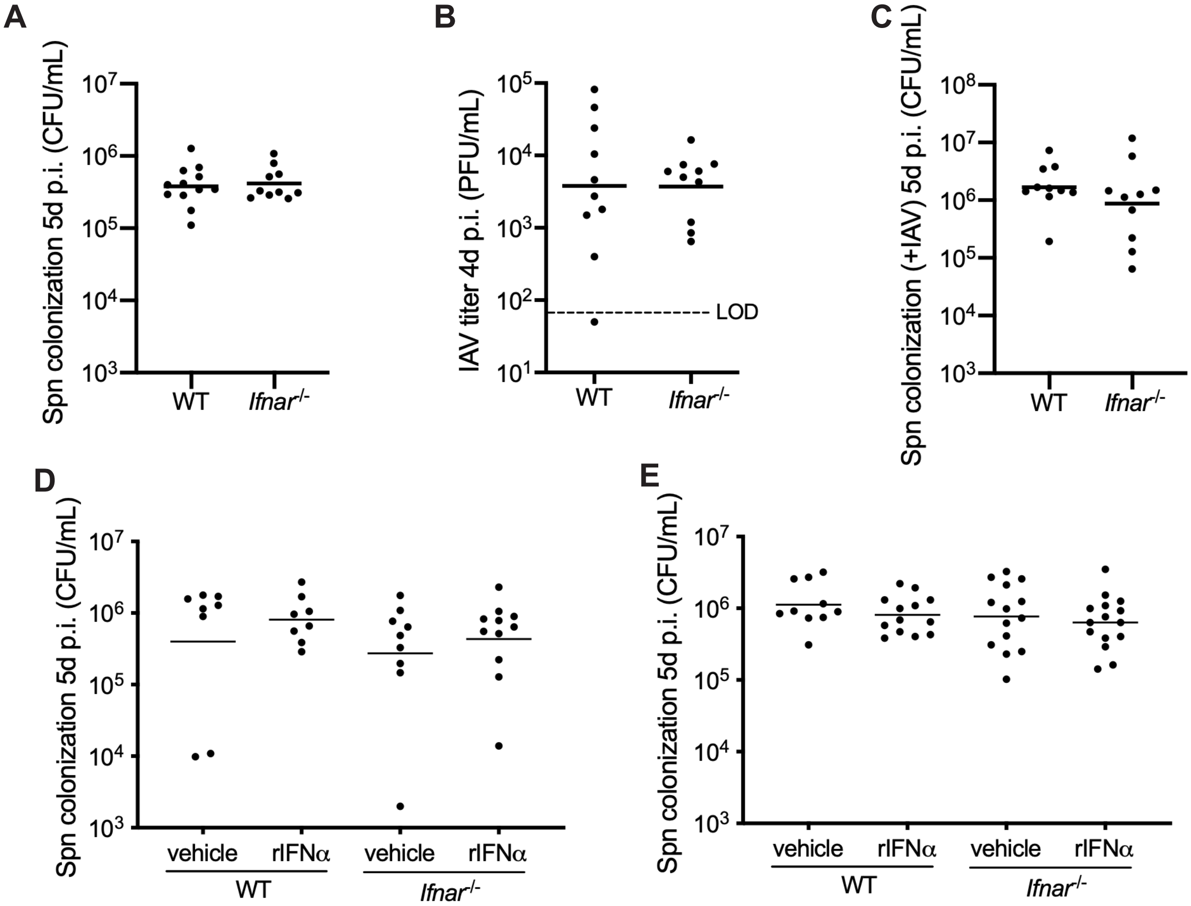
A
Spn colonization 5d p.i. (CFU/mL)
10^7
10^6
10^5
10^4
10^3
WT
Ifnar-/-
B
IAV titer 4d p.i. (PFU/mL)
10^5
10^4
10^3
10^2
10^1
LOD
WT
Ifnar-/-
C
Spn colonization (+IAV) 5d p.i. (CFU/mL)
10^8
10^7
10^6
10^5
10^4
10^3
WT
Ifnar-/-
D
Spn colonization 5d p.i. (CFU/mL)
10^7
10^6
10^5
10^4
10^3
vehicle
rIFNα
vehicle
rIFNα
WT
Ifnar-/-
E
Spn colonization 5d p.i. (CFU/mL)
10^7
10^6
10^5
10^4
10^3
vehicle
rIFNα
vehicle
rIFNα
WT
Ifnar-/-

Supplemental Figure 1. Spn colonization and IAV titer does not differ in WT and *Ifnar1*$^{-/-}$ pups. Pups were infected IN with either 10^3 CFU Spn or 250 PFU IAV-x31. A) Spn URT colonization was not different between WT and *Ifnar1*$^{-/-}$ pups. B) There was no difference in viral titer in the URT of WT and *Ifnar1*$^{-/-}$ pups. C) WT and *Ifnar1*$^{-/-}$ pups first received IAV and then Spn; bacterial colonization was not different between pups. D and E: WT and *Ifnar1*$^{-/-}$ pups were infected IN with 10^3 CFU Spn and daily received 1000 IU of recombinant mouse IFNα2, or 1000-5000 IU of recombinant mouse IFNβ, or vehicle control (0.1%BSA-PBS) by IN instillation. Treatment of WT pups with rIFNα2 (D) or rIFNβ (E) did not affect bacterial colonization.

Colonization and titer data are for individual pups with the line indicating the geometric mean. Each symbol represents the value from an individual pup on a single day. n ≥ 8 pups/group. ns, not significant (Mann-Whitney test). Dotted line shows limit of detection.
