# Supplementary material for: Type I Interferon Signaling Is a Common Factor Driving Streptococcus pneumoniae and Influenza A Virus Shedding and Transmission
Source: mBio. 2021 Feb 16;12(1):e03589-20. doi: 10.1128/mBio.03589-20 (PMC8545127; doi:10.1128/mBio.03589-20)
Supplement: FIG S2 [file mbio.03589-20-sf002.pdf]

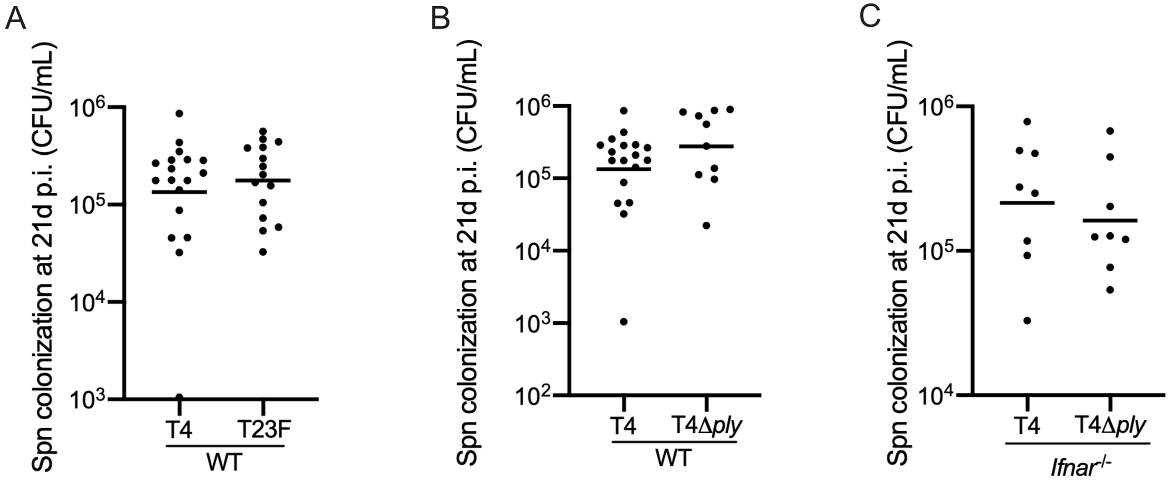
A
B
C
Spn colonization at 21d p.i. (CFU/mL)
10^6
10^5
10^4
10^3
10^2
T4
T23F
WT
T4Δply
Ifnar-/-

Supplemental Figure 2. Spn T4 and T23F colonization at 21d p.i. does not differ in WT and *Ifnar1*$^{-/-}$ mice. A) WT pups were infected IN with 10^3 CFU Spn T4 or T23F or (B and C) WT and *Ifnar1*$^{-/-}$ pups were infected IN with 10^3 CFU Spn T4 or an isogenic T4 *ply*$^-$ mutant; colonization was assessed 21d p.i. A) The mice are still highly-colonized with Spn of both T4 and T23F serotypes. B) Colonization of T4 and the isogenic T4 *ply*$^-$ mutant is not different. C) At 21d p.i., there is no difference in the colonization between T4 and T4 *ply*$^-$ strains in *Ifnar1*$^{-/-}$ mice. Colonization data are for individual pups with the line indicating the geometric mean. Each symbol represents the value from an individual pup on a single day. n ≥ 8 pups/group. (Not significant, Mann-Whitney test).
