# Supplementary material for: Type I Interferon Signaling Is a Common Factor Driving Streptococcus pneumoniae and Influenza A Virus Shedding and Transmission
Source: mBio. 2021 Feb 16;12(1):e03589-20. doi: 10.1128/mBio.03589-20 (PMC8545127; doi:10.1128/mBio.03589-20)
Supplement: FIG S3 [file mbio.03589-20-sf003.pdf]

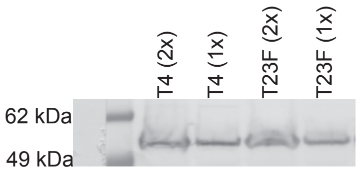
T4 (2x)
T4 (1x)
T23F (2x)
T23F (1x)
62 kDa
49 kDa

Supplemental Figure 3. Immunoblot of Spn strains for pneumolysin. Spn serotypes T4 and T23F used in this study do not produce different levels of pneumolysin.
