# Supplementary material for: Type I Interferon Signaling Is a Common Factor Driving Streptococcus pneumoniae and Influenza A Virus Shedding and Transmission
Source: mBio. 2021 Feb 16;12(1):e03589-20. doi: 10.1128/mBio.03589-20 (PMC8545127; doi:10.1128/mBio.03589-20)
Supplement: FIG S4 [file mbio.03589-20-sf004.pdf]

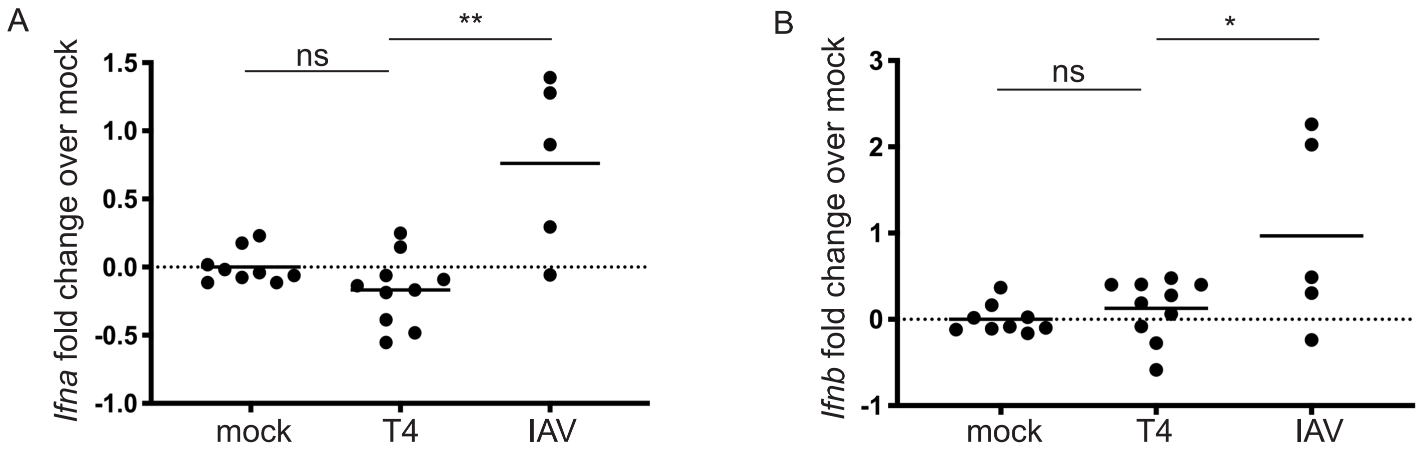
A
B
Ifna fold change over mock
Ifnb fold change over mock
ns
**
*
1.5
1.0
0.5
0.0
-0.5
-1.0
3
2
1
0
-1
mock
T4
IAV

Supplemental Figure 4. Increased expression of *Ifna* and *Ifnb* was not detected at 48h p.i. in mice that had received Spn. WT pups were infected IN with 10^3 CFU Spn T4 or 250 PFU IAV; mock mice received PBS. RNA was isolated from URT lavages 48h p.i. and analyzed by qRT-PCR. Spn-infected mice showed no increased expression of *Ifna* or *Ifnb* over mock mice. This increased expression was seen only in IAV-infected mice. Gene expression data are $\log_{10}$ transformed; each symbol represents the value from an individual pup. Comparisons (Mann-Whitney test) are to mock mice. $n \geq 5$ pups/group. ns, not significant; **, $P < 0.01$.
