# Supplementary material for: Type I Interferon Signaling Is a Common Factor Driving Streptococcus pneumoniae and Influenza A Virus Shedding and Transmission
Source: mBio. 2021 Feb 16;12(1):e03589-20. doi: 10.1128/mBio.03589-20 (PMC8545127; doi:10.1128/mBio.03589-20)
Supplement: TABLE S3 [file mbio.03589-20-st003.docx]

**Supplemental Table 3.** Primers used in the study.

| Target gene | Forward primer | Reverse primer |
| --- | --- | --- |
| *Gapdh* | 5’ TGTGTCCGTCGTGGATCTGA 3’ | 5’ CCTGCTTCACCACCTTCTTGA 3’ |
| *Ifna* | 5’ TCTGATGCAGCAGGTGGG 3’ | 5’ AGGGCTCTCCAGACTTCTGCTCTG 3’ |
| *Ifnb* | 5’ AGACTATTGTTGTACGTCTCC 3’ | 5’ CAGTAATAGCTCTTCAAGTGG 3’ |
| *Ifit2* | 5’ AGTACAACGAGTAAGGAGTCACT 3’ | 5’ AGGCCAGTATGTTGCACATGG 3’ |
| *Mx1* | 5’ TCTGAGGAGAGCCAGACGAT 3’ | 5’ ACTCTGGTCCCCAATGACAG 3’ |
| *Oasl2* | 5’ GGATGCCTGGGAGAGAATCG 3’ | 5’ TCGCCTGCTCTTCGAAACTG 3’ |
| *St3gal1* | 5’ GGTTCCCCAAGCAGATGCTC 3’ | 5’ CCAATATGATACCTTGTCCTGGC 3’ |
